# TLR2-Modulating Lipoproteins of the *Mycobacterium tuberculosis* Complex Enhance the HIV Infectivity of CD4+ T Cells

**DOI:** 10.1371/journal.pone.0147192

**Published:** 2016-01-25

**Authors:** Ciaran Skerry, Lee G. Klinkenberg, Kathleen R. Page, Petros C. Karakousis

**Affiliations:** 1 Department of Medicine, Johns Hopkins University School of Medicine, Baltimore, Maryland, United States of America; 2 Department of International Health, Johns Hopkins Bloomberg School of Public Health, Baltimore, Maryland, United States of America; Fundació Institut d’Investigació en Ciències de la Salut Germans Trias i Pujol, Universitat Autònoma de Barcelona, SPAIN

## Abstract

Co-infection with *Mycobacterium tuberculosis* accelerates progression from HIV to AIDS. Our previous studies showed that *M*. *tuberculosis* complex, unlike *M*. *smegmatis*, enhances TLR2-dependent susceptibility of CD4+ T cells to HIV. The *M*. *tuberculosis* complex produces multiple TLR2-stimulating lipoproteins, which are absent in *M*. *smegmatis*. *M*. *tuberculosis* production of mature lipoproteins and TLR2 stimulation is dependent on cleavage by lipoprotein signal peptidase A (LspA). In order to determine the role of potential TLR2-stimulating lipoproteins on mycobacterial-mediated HIV infectivity of CD4+ T cells, we generated *M*. *smegmatis* recombinant strains overexpressing genes encoding various *M*. *bovis* BCG lipoproteins, as well as a *Mycobacterium bovis* BCG strain deficient in LspA (Δ*lspA*). Exposure of human peripheral blood mononuclear cells (PBMC) to *M*. *smegmatis* strains overexpressing the BCG lipoproteins, LprF (p<0.01), LprH (p<0.05), LprI (p<0.05), LprP (p<0.001), LprQ (p<0.005), MPT83 (p<0.005), or PhoS1 (p<0.05), resulted in increased HIV infectivity of CD4+ T cells isolated from these PBMC. Conversely, infection of PBMC with Δ*lspA* reduced HIV infectivity of CD4+ T cells by 40% relative to BCG-infected cells (p<0.05). These results may have important implications for TB vaccination programs in areas with high mother-to-child HIV transmission.

## Introduction

Despite significant improvements in expanding access to global HIV prevention and treatment services in the last decade, an estimated 2 million people were newly infected with HIV in 2014 [1]. A majority of these new infections occurred in tuberculosis-endemic countries, where BCG vaccination at birth is virtually universal. The success of effective behavioral interventions to prevent HIV infection, such as condom use, has been limited by suboptimal uptake in at-risk populations. Universal treatment of all HIV-infected individuals with antiretroviral therapy (ARV) could halt new transmission, but identifying, linking and retaining most HIV-infected individuals in care is challenging [2]. Biological determinants of HIV susceptibility are complex and not fully understood, but could improve HIV prevention strategies. For example, the removal of HIV target cells in the male foreskin through circumcision is a simple one-time procedure that reduces the risk of HIV by 60% [3]. Treatment of sexually transmitted infections also reduces HIV risk through a variety of mechanisms, such as restoring integrity of the mucosal barrier, and reducing the number of activated immune target cells at sites of HIV exposure [4,5,6]. Minimizing inflammation is important because activated CD4^+^ T cells are much more susceptible to infection than resting T cells [7,8].

HIV pathogenesis and susceptibility is associated with immune activation [9]. HIV preferentially infects activated T cells, which compared to quiescent cells, express a large number of host factors required for HIV replication [10,11]. Co-infection with pathogens common in HIV-endemic areas has been postulated to have an impact on HIV susceptibility [7,12]. Compared to Europeans, Africans and Asians have elevated systemic immune activation markers, which could drive regional disparities in HIV rates around the world [12,13]. Using a single cycle HIV infectivity assay, we have shown that *ex vivo* infection of peripheral blood mononuclear cells (PBMC) with *Mycobacterium tuberculosis* or *M*. *bovis* BCG (MTB complex), but not with *M*. *smegmatis*, enhances susceptibility of CD4+ T cells to HIV infection independent of T cell activation state. Cells exposed to *M*. *smegmatis* were found to express lower levels of TLR2, and their ability to become infected with R5-tropic pseudovirus was reduced upon siRNA-mediated silencing of TLR2 [14]. In TB infection, lipoproteins are important mediators of TLR2 activation. The lipoprotein signal peptidase, LspA, is an important virulence factor necessary for mycobacterial production of mature lipoproteins [15,16]. In the absence of LspA, cleavage of the conserved N-terminal “lipobox” of prolipoproteins does not occur, preventing them from being processed into mature lipoproteins [16,17,18]. Here, we show that the enhanced HIV infectivity of CD4+ T cells derived from *M*. *bovis* BCG-infected PBMC requires LspA. Furthermore, the overexpression of *M*. *bovis* BCG-derived lipoproteins, including LprF, LprH, LprI, LprP, LprQ, MPT83, and PhoS1, by *M*. *smegmatis* results in significantly greater *ex vivo* HIV infection of human PBMC-derived CD4+ T cells. The ability of these lipoproteins to increase the HIV infectivity of CD4+ cells is reversed by chemical inhibitors of TLR2 signaling.

## Results

### Overexpression of *M*. *bovis* BCG lipoproteins increases HIV susceptibility of CD4+ T cells derived from *M*. *smegmatis*-infected human PBMC

**Fig 1:**
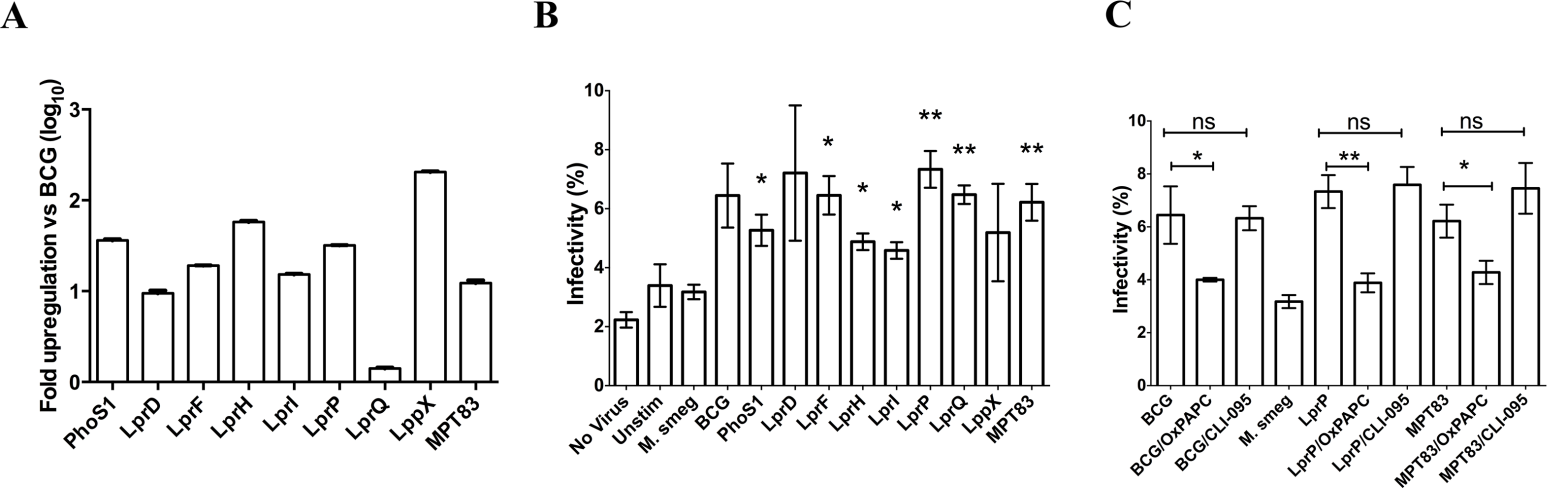
Overexpression of *M*. *bovis* BCG lipoproteins increases HIV susceptibility of *M*. *smegmatis*-infected human CD4+ T cells. A. Levels of expression of *M*. *bovis* BCG lipoprotein genes in various *M*. *smegmatis* knock-in strains. Cycle threshold values were first normalized to the housekeeping gene, *sigA*, and expressed as fold increase relative to wild-type *M*. *bovis* BCG. Each cycle difference was assumed to represent a 2-fold difference in gene expression. The data are representative of three independent experiments. B. The percentage of X4-tropic, GFP-expressing pseudovirus-infected CD4+ cells without any stimulation (Unstim) or after infection with *M*. *bovis* BCG Copenhagen (BCG), *M*. *smegmatis* MC^2^155 containing empty vector (M. smeg), or *M*. *smegmatis* strains overexpressing BCG lipoproteins PhoS1, LprQ, LprD, LprI, LprH, LprF, LppX, LprP, or MPT83. C. The TLR2 agonist zymosan (Zym), TLR2 and TLR4 antagonist OxPAPC, or the TLR4-specific antagonist CLI-095 was added to PBMC prior to HIV infection. Results represent combined flow cytometric analysis of CD4+ cells after infection with an X4-tropic GFP+ pseudovirus, from three independent experiments. * p< 0.05; ** p< 0.005.

*M*. *bovis* BCG infection of PBMC increases the HIV infectivity of CD4+ T cells, and this phenomenon has been attributed to TLR2 induction [14]. *M*. *bovis* BCG lipoproteins are important TLR2 agonists [19,20,21]. We hypothesized that lipoproteins expressed in *M*. *tuberculosis* and *M*. *bovis* BCG (MTB complex), but not in *M*. *smegmatis*, are responsible for the increased HIV infectivity of CD4+ T cells following MTB complex infection of PBMC *ex vivo*. The following lipoprotein genes present in *M*. *bovis* BCG, but absent in *M*. *smegmatis*, were cloned into *M*. *smegmatis* MC^2^155 under control of the constitutively active *hsp60* promoter: *lppX*, *lprD*, *lprF*, *lprH*, *lprI*, *lprP*, *lprQ*, *mpt83*, and *phoS1* (Table 1). Production of these lipoproteins under the control of the constitutive *hsp60* promoter resulted in variable expression levels. Each lipoprotein-encoding gene was expressed at higher levels in the corresponding knock-in strain relative to *M*. *bovis* BCG (Fig 1A). Seven of the nine lipoprotein knock-in mutants induced greater HIV infectivity of CD4+ T cells than wild-type *M*. *smegmatis*: *lprF* (2.0-fold; p<0.01); *lprH* (1.5-fold; p<0.05); *lprI* (1.4-fold; p<0.05); *lprP* (2.3-fold; p<0.001); *lprQ* (2.0-fold (p<0.005), *mpt83* (2.0 fold; p<0.005); and *phoS1* (1.7-fold; p<0.05). However, *lppX* and *lprD* knock-in strains failed to increase infectivity beyond empty-vector controls (Fig 1B). Addition of the dual TLR2/TLR4 inhibitor, OxPAPC, significantly reduced the HIV infectivity of CD4+ T cells following infection of PBMC with *M*. *bovis* BCG, while addition of the TLR4 inhibitor, CLI-095, showed no effect (Fig 1C), confirming our previous findings that *M*. *bovis* BCG infection of PBMC increases the HIV infectivity of CD4+ T cells through a TLR2-dependent mechanism [14]. Addition of the TLR2 agonist zymosan to *M*. *smegmatis* cultures prior to PBMC infection increased HIV infectivity of isolated CD4+ T cells 6.2-fold relative to untreated *M*. *smegmatis* cultures (p<0.01; Fig 1C). Whereas the addition of OxPAPC to the *M*. *smegmatis* knock-in strains overexpressing *lprP* or *mpt83* during PBMC infection reversed the enhanced HIV infectivity of isolated CD4+ T cells to levels similar to those following infection with wild-type *M*. *smegmatis*, addition of CLI-095 had no such effect on PBMC infected with the *mpt83* knock-in strain (Fig 1C). OxPAPC alone did not alter HIV uptake by CD4+ T cells uninfected by mycobacteria (data not shown). Taken together, these results support our hypothesis that various *M*. *tuberculosis*-specific lipoproteins may contribute to the *M*. *bovis* BCG-mediated increase in HIV infectivity of CD4+ T cells and this phenomenon may be inhibited by the use of TLR2 antagonists.

**Table 1:** List of gene-specific primer pairs used to generate mycobacterial recombinant strains.

Gene	Fwd Primer	Rv Primer
*phoS1*	gatacgtcgccggactgt	gtcaacgaggctagctggaa
*lppX*	gtgagcgcaaatgaatgatg	aattttcagatgccgtcagg
*mpt83*	actccgaatcactggtgtcc	gtgatctatcggcacgaagc
*lprH*	ctcgttgctggctcgatac	ccgtgggacacatttgagta
*lprD*	cacctggggtattaggcaag	ggtgcgaatcctctcgac
*lprF*	atggacggggctggattc	ggttcgcgcctgttatcc
*lprI*	accagtctgagccctgtgg	gcacgcgctaaagatattgc
*lprQ*	tgcatgttgtagctgcgttt	ccttgaccagatcgtcatcc
*lprP*	acccgaactatcacccatga	gatcggatggccttttgtt

All primers are designed to function in the Gateway system (start with GGGG-AttB1-MCS-gene specific primer).

### Deficiency of lipoproteins reverses *M*. *bovis* BCG-mediated induction of HIV infectivity of CD4+ T cells

**Fig 2:**
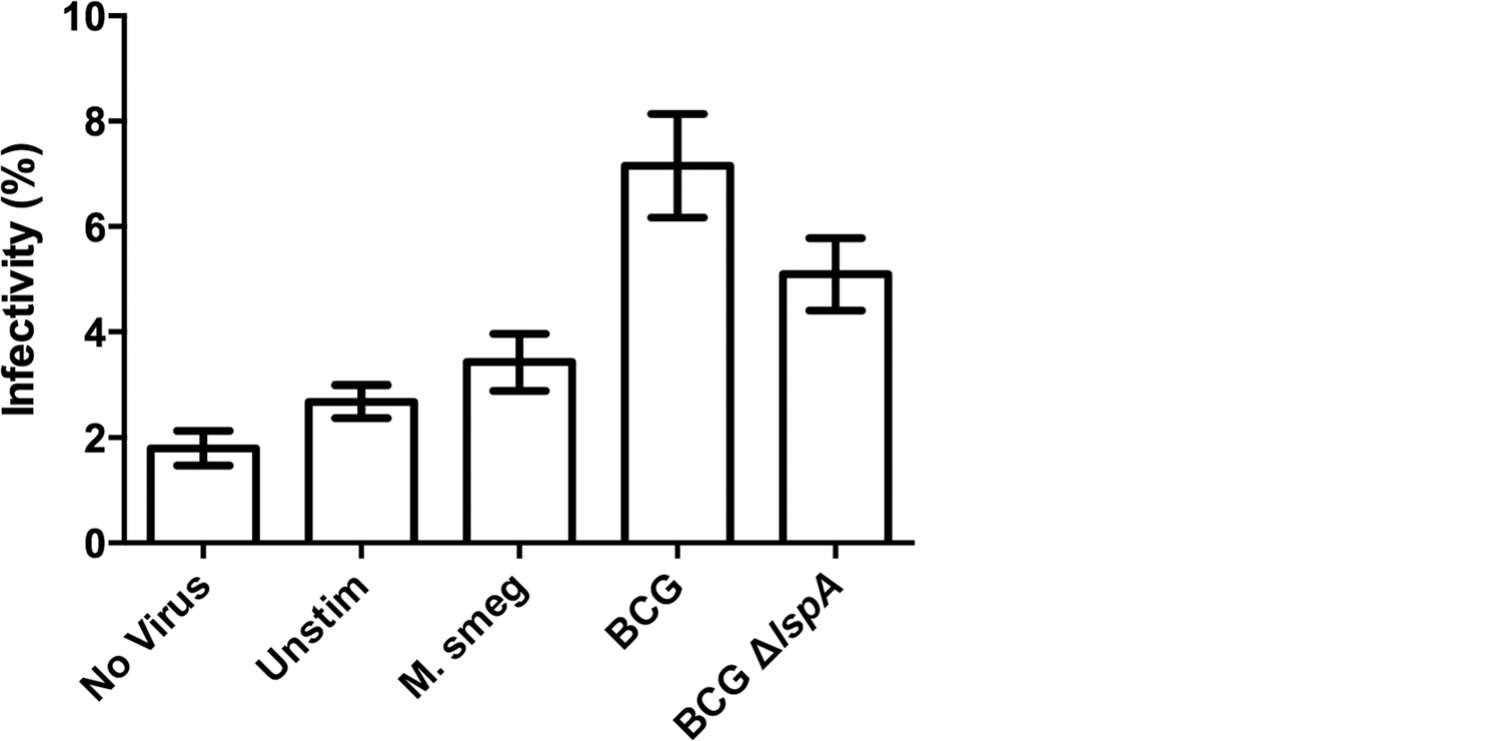
Deficiency of LspA partially reverses *M*. *bovis* BCG-mediated induction of HIV infectivity of CD4+ T cells. The percentage of GFP+ CD4+ T cells following no stimulation (Unstim), or stimulation of PBMC with *M*. *bovis* BCG Copenhagen (BCG), *M*. *smegmatis* MC^2^155 containing empty vector (M. smeg), or a *M*. *bovis* BCG strain lacking lipoprotein signal peptidase A (BCGΔ*lspA*), which fails to produce mature lipoproteins. Results represent combined flow cytometric analysis of CD4+ cells after infection with an X4-tropic GFP+ pseudovirus, from 3 independent experiments. * p< 0.05

LspA cleaves the signal peptide from the C-terminus of mycobacterial proteins during maturation [16]. Mycobacterial strains lacking LspA fail to express mature lipoproteins and exhibit impaired TLR2 signaling [15,16]. We generated a *M*. *bovis* BCG strain deficient in the *lspA* gene (Δ*lspA*) using a targeted suicide plasmid [22]. Pre-infection of PBMC with Δ*lspA* resulted in significantly reduced HIV infectivity of isolated CD4+ T cells relative to those infected with wild-type *M*. *bovis* BCG (Fig 2, p = 0.013), although HIV infectivity of CD4+ T cells following PBMC infection with the mutant remained significantly higher than that observed following that with wild-type *M*. *smegmatis* (p<0.01).

## Discussion

Although vaccination with *M*. *bovis* BCG is contraindicated in HIV-infected individuals due to the risk of disseminated disease [23], this vaccine continues to be administered around the world to neonates at risk for acquiring HIV through contaminated breast milk of HIV-infected mothers. Therefore, a deeper understanding of how *M*. *bovis* BCG infection enhances the HIV infectivity of CD4+ T cells may lead to safer TB vaccines, especially in HIV co-endemic areas. Previous work from our group identified a TLR2-mediated increase in HIV infectivity of CD4+ T cells following infection of PBMC with *M*. *bovis* BCG compared to *M*. *smegmatis* [14]. This phenomenon was independent of T cell activation markers CD38 and HLA-DR. Here, we further elucidate the mechanism of increased infectivity, identifying a key role for lipoproteins. Interestingly, although *M*. *smegmatis* expresses several TLR2-stimulating lipoproteins and LspA [24], these do not appear to modulate CD4+ T cell-HIV interactions in the same way as their *M*. *tuberculosis* complex counterparts. Thus, the overexpression of *M*. *bovis* BCG lipoproteins in *M*. *smegmatis* leads to increased HIV infectivity of PBMC-derived CD4+ T cells. Conversely, the infection of PBMC with a strain of *M*. *bovis* BCG unable to produce mature lipoproteins resulted in significantly reduced HIV infectivity of CD4+ T cells.

We chose to evaluate a Δ*lspA M*. *bovis* BCG mutant for several practical reasons. First, there is significant redundancy in TLR2 signaling by mycobacterial lipoproteins, such that disrupting individual lipoproteins may not impact *ex vivo* HIV susceptibility. Because LspA is required for cleavage of prolipoprotein signal peptides prior to trafficking of all lipoproteins to the cell wall, the Δ*lspA M*. *bovis* BCG mutant allowed for a more comprehensive assessment of the role of lipoproteins on HIV susceptibility of CD4+ T cells. LspA-deficient *M*. *tuberculosis* mutants lack cell wall-associated lipoproteins and exhibit decreased TLR2 agonist activity [15,16]. In addition, LspA is required for full *M*. *tuberculosis* virulence in macrophages and in the mouse model [16]. However, further studies are required to evaluate the immunogenicity of the Δ*lspA M*. *bovis* BCG mutant relative to the wild-type strain.

There are several important limitations to our study. While disruption of lipoprotein processing reduced HIV infectivity of CD4+ T cells, this reduction did not achieve levels observed following exposure to *M*. *smegmatis*. This suggests that there are non-lipoprotein factors influencing the BCG-mediated increase in HIV infectivity of CD4+ T cells. Glycolipids of the mycobacterial cell wall, particularly phosphatidylinositol mannoside 6 (PIM6) [25], have also been implicated in enhancing TLR2-mediated HIV replication in T cells and may be important to explore in our model. It should also be noted that the *M*. *smegmatis* lipoprotein knock-in strains used in the current study express these lipoproteins at much higher levels than does wild-type *M*. *bovis* BCG, and we cannot implicate a specific role for any individual lipoprotein. It is highly likely that *M*. *tuberculosis* complex-specific lipoproteins and LspA modulate interactions between HIV and CD4+ T cells beyond simply stimulating TLR2. For example, an *lspA*-deficient *M*. *tuberculosis* mutant showed impaired resistance to malachite green, suggesting a cell wall permeability defect [REF], which might alter macrophage functions in a TLR2-independent manner and reduce susceptibility of CD4+ T cells to HIV infection. Furthermore, growth kinetics and other, non-lipoprotein-related genetic differences between the nonpathogenic *M*. *smegmatis* and *M*. *bovis* BCG may influence HIV infectivity of CD4+ T cells through differential induction of host defense mechanisms, such as cytokine production and cell death pathways. Finally, it is difficult to extrapolate our findings from an *ex vivo* model of HIV susceptibility to HIV risk in human populations. Interestingly, recent work suggests that immunization of infant rhesus macaques with *M*. *bovis* BCG renders them more susceptible to SIV infection following repeated challenge (Kristina De Paris, personal communication). Given that BCG is the most commonly used vaccine worldwide [26], even a small effect on maternal-to-child transmission of HIV could be of public health significance.

Our study demonstrates that lipoproteins expressed by virulent mycobacteria may activate immune pathways that enhance T-cell susceptibility to HIV. Future studies will focus on extending our *ex vivo* observations to clinically relevant animal models, such as humanized mice and nonhuman primates, as well as testing the protective efficacy of *M*. *bovis* BCG Δ*lspA* against aerosol challenge with virulent *M*. *tuberculosis* relative to the wild-type and complemented strains in guinea pigs. Finally, TLR2-independent pathways responsible for the *M*. *bovis* BCG-mediated increase in HIV infectivity of CD4+ T cells will be further explored. Given the magnitude of the TB epidemic, particularly in the regions of the world with the highest rates of HIV, these results highlight the need to measure HIV risk when evaluating the safety of TB vaccines at a population level.

## Methods

### Ethics Statement

Written informed consent was obtained from all subjects prior to entry into the study. The study was approved by the Johns Hopkins University School of Medicine Institutional Review Board. All studies were conducted in accordance with institutional and federal guidelines and regulations on human subjects research.

### Subjects

HIV-seronegative healthy individuals (ages 25–35) without prior history of BCG vaccination or TB infection were recruited at Johns Hopkins University School of Medicine. All subjects were confirmed to have negative tuberculin skin tests prior to entry into the study.

### Cell Isolation and Culture

Peripheral blood mononuclear cells (PBMC) were isolated from healthy volunteers and antigenic stimulation of cells was performed by incubating cells with whole bacteria or antigen for 48–72 hours at 37°C with 5% CO_2_. For infectivity assays, unstimulated cells were used as negative control and PHA (50 μg/ml) stimulated cells were used as positive control to ensure that cells were responsive to *in vitro* stimulation. Inhibition of TLR signaling was achieved through incubation of PBMC with OxPAPC or CLI-095 (40 μg/ml).

### Bacterial Strains and Mycobacterial Infection

*M*. *bovis* BCG (Copenhagen), *M*. *tuberculosis* CDC1551, and *M*. *smegmatis* MC^2^155 strains procured from ATCC were used for stimulation of PBMC at an MOI of 1:4. The bacterial strains were grown to mid-logarithmic phase in 7H9 broth (Difco, Sparks, MD) supplemented with 10% oleic acid-albumin-dextrose-catalase (OADC, Difco), glycerol, 0.05% Tween 80 at 37°C on a shaker. At the time of stimulation, bacteria were pelleted and resuspended in complete RPMI and co-cultured with isolated PBMC in complete RPMI supplemented with IL-2 at 37°C for 24–48 hours.

### Single-cycle HIV-1 Infection Assay

After stimulation, the cells were washed with fresh RPMI and CD4+ cells were isolated from stimulated PBMC by negative selection using a CD4+ T cell isolation kit (Miltenyi biotech; GmbH) according to the manufacturer’s instructions. Isolated CD4+ cells were infected with a single-cycle replication-competent, green fluorescent protein (GFP)-expressing proviral construct pseudotyped with CCR5-tropic or CXCR4-tropic envelope (NL43-deltaEnvGFP), as previously described [27]. Briefly, stimulated CD4+ cells were spinoculated for 2 hrs with the virus. The infected cells were incubated at 37°C for 72 hours. Fluorescence emitted by the cells carrying GFP-expressing virus was quantified using Cellquest Pro software (Beckton Dickinson) to calculate percent infectivity.

### Immunoflourescence Staining and Flow Cytometry Analysis

Stimulated CD4+ cells were washed and then stained with directly conjugated antibodies to cell surface markers, including CD4-FITC, CD3-PE, CD38-PE, HLA DR-FITC, CD195–PE, and CD184-FITC (Becton Dickinson, San Jose, CA) and TLR-2 FITC (R&D biosystems). Marker expression analysis was performed using a FACSCalibur flow cytometer (BDIS). A minimum of 10,000 viable lymphocytes was collected for each run.

### Construction of mutant strains

*M*. *smegmatis* strains overexpressing individual lipoproteins were generated in the pGS400H plasmid backbone [28] using the Gateway system (Invitrogen, USA). This construct placed the coding sequence of the lipoprotein gene under the control of a constitutive Hsp60 promoter [29]. These constructs contain a hygromycin selectable marker and integration occurred at the attB site as a single copy. The gene-specific primers pairs used for cloning are listed in Table 1.

The *M*. *bovis* BCG strain lacking *lspA* was generated using a conditionally replicating mycobacteriophage, as previously described [30]. Briefly, Upper (886 bp) and lower (1125bp) allelic exchange substrates (AES) were amplified from CDC1551 and *M*. *bovis* BCG genomic DNA before ligation into pCR2.1-TOPO. Following sequencing, AES was subcloned into pYUB854. After transduction, BCG was plated onto Middlebrook 7H10 agar with ADC and hygromycin (50 μg/ml) and colonies screened for the absence of *lspA*.

### Statistical Analysis

Statistical significance was determined by students T Test. A p-value of < .05 was considered statistically significant.
